# A broadband second-order bandpass frequency selective surface for microwave and millimeter wave application

**DOI:** 10.1038/s41598-024-62228-3

**Published:** 2024-05-27

**Authors:** Zhiming Li, Xiaolong Weng, Xu Yi, Kai Li, Wei Duan, Mei Bi

**Affiliations:** 1 School of Electronic Science and Engineering, University of Electronic Science and Technology of China, Chengdu, 611731 China; 2 School of Materials and Energy, University of Electronic Science and Technology of China, Chengdu, 611731 China

**Keywords:** Metamaterials, Composites, Sub-wavelength optics

## Abstract

This paper presents a frequency selective surface (FSS) with a wideband second-order bandpass response in the dual-band of microwave and millimeter wave. The overall structure consists of three layers of metal pattern and two layers of thin dielectric substrate. The top and bottom metal layers have capacitive patches with integrated curled Jerusalem cross slot resonators, while the intermediate metal layer has an inductive grid structure with cross-shaped slot resonators. The incorporated slot resonators play a pivotal role in achieving the desired transmission poles or zeros, which enable a wideband second-order filtering response in the dual-band and a quick roll-off at the passband edges, increasing the efficacy of electromagnetic shielding. To fully investigate the structure's frequency response, an equivalent circuit model of the structure is created, spanning the complete frequency range of 5–50 GHz. Physical samples are created and measured to confirm the suggested approach’s efficacy. The passband center frequencies of the FSS are found at *f*_1_ = 19.42 GHz and *f*_2_ = 42.78 GHz, and the − 3 dB bandwidth is 4.34 GHz (17.25–21.59 GHz) and 8.54 GHz (38.51–47.05 GHz), respectively. The simulation results align well with the experimental data. The transmission response rapidly transitions from the passband to the stopband at the passband boundaries.

## Introduction

When interacting with electromagnetic waves, the FSS can display specific bandpass or bandstop filtering characteristics and serve as a spatial filter in two dimensions^1,2^. The FSS has become widely used in a number of fields recently, including aircraft antenna radomes^3–7^, satellite communications^8–11^, electromagnetic shielding^12–16^, and microwave absorbers^17–19^. Detection radars have progressively extended their operational frequencies to the microwave and millimeter-wave dual-bands, accompanied by substantially higher bandwidths and data transmission rates, due to the rapid advancement of radar systems and communication technologies^20,21^. A FSS with wideband, wide-angle, polarization-insensitive characteristics and high out-of-band suppression is therefore urgently needed. This will ensure the antenna's electromagnetic transparency within its diverse operational frequency bands while decreasing its electromagnetic interference outside of those bands.

Traditionally, achieving multi-band FSS has involved the use of multi-resonant units^22–25^, complementary structures^26,27^, and fractal structure designs^28,29^. In reference^30^, a two-dimensional double annular periodic array and a grid array are cascaded to create a dual-band FSS. This FSS exhibits good angular stability and wideband transmission in the X-band and Ka-band frequency ranges. A three-dimensional (3D)-FSS that has efficient transmission and out-of-band suppression performance for signals in the S-band and Ku-band is described in reference^31^. This was accomplished by constructing 3D resonant cavities using a particular configuration of a two-dimensional periodic array, which permits two distinct propagation modes. The design of a dual-band FSS utilizing quarter-mode substrate integrated waveguide technology is presented in Reference^32^. The FSS demonstrates a quasi-elliptic passband frequency response, effectively reducing structure thickness and achieving strong out-of-band suppression. The passband bandwidth and interband shielding performance need to be enhanced for practical applications. In reference^33,34^, the dual-band FSSs with strong out-of-band suppression and high-order bandpass response characteristics are designed based on the AC-DMPR aperture-coupled dual-mode patch resonator. This design significantly expands the frequency-band interval while maintaining a low-profile structure. In addition, it is a challenge for dual-band FSS design to achieve broadband operating frequency, strong out-of-band shielding, good polarization, and angular stability at the same time^14,35–40^.

In this paper, a novel second-order band-pass FSS is introduced that operates in microwave and millimeter wave bands and has wide bandwidth and high angular stability. The proposed FSS is a multilayer structure consisting of periodic metallic layers with effective inductance, capacitance, and resonant surface impedance, along with two thin dielectric substrates. In this study, a cross-shaped slot and a Jerusalem cross slot are embedded in metallic layer to broaden the passband and stopband, and improve the electromagnetic shielding effectiveness out-of passband. The FSS achieves a − 3 dB passband range of 17.25–21.59 GHz and 38.51–47.05 GHz, respectively. An ECM is used to analyze the working mechanism of the structure, and experimental validation is performed. The measurement results demonstrate excellent stability of the FSS under TE and TM polarizations when the incident angle varies from 0° to 60°. These results indicate that the proposed FSS is a promising candidate for dual-band radar communication in the microwave and millimeter wave domains.

## Methods

### Structure of the proposed FSS

**Figure 1:**
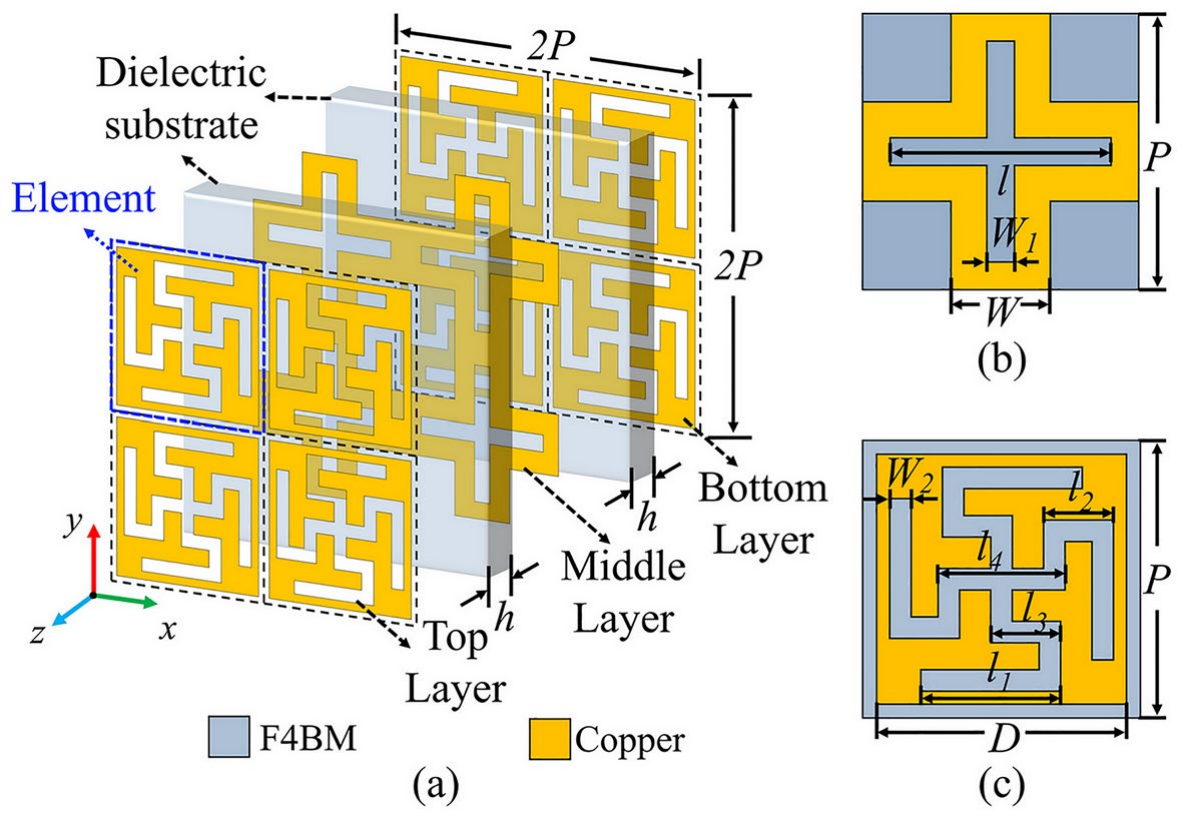
The structure of the dual-band FSS, (**a**) Three-dimensional perspective of the proposed FSS, (**b**) Middle layer FSS component, (**c**) Top and bottom FSS components.

The three-dimensional topology of the proposed dual-band FSS is shown in Fig. 1a. Two thin dielectric substrates separate the upper, middle, and lower three metal layers. As shown in Fig. 1b, the middle layer contains a metallic inductive grid with a cross-shaped slot and the length *l* and width *W*_1_ specifications. The unit structure, which is defined by a metallic capacitive patch with a curled cross-shaped slot, is identical in the top and bottom layers, as illustrated in Fig. 1c. The F4B-M dielectric substrate has a thickness of *h* = 0.45 mm, relative permittivity *ε*_*r*_ = 2.65, and loss tangent *tanδ* = 0.0013. The non-resonant cell structure composed of the capacitor patch, inductance grid, and capacitor patch can effectively realize the first passband with second-order band-pass filtering characteristics. In order to achieve dual-band transmission performance, the cross-shaped slot resonator and curled slot resonator are integrated in the non-resonant unit structure. This integration allows for the establishment of a second passband while maintaining the original wideband transmission properties. The refined structural parameters are detailed in Table 1.

**Table 1:** Geometric parameters of the proposed FSS cells.

Parameter	Dimensions (mm)	Parameter	Dimensions (mm)
*P*	2.3	*D*	2.08
*l*	1.84	*l*_1_	1.0
*l*_2_	0.54	*l*_3_	0.54
*l*_4_	1.0	*W*	1.1
*W*_1_	0.2	*W*_2_	0.2

### Equivalent circuit model (ECM)

A FSS unit exposed to an electromagnetic wave can be conceptualized physically as a resonant circuit. Combinations of capacitance and inductance are efficient representations of the electrical behavior of FSS. The analogous circuit method can be used to gain a deeper grasp of the FSS's operational principles. The following expression can be used to calculate the resonant frequency *f*:

1 $$f = \frac{1}{{2\pi \sqrt {L_{E} C_{E} } }}$$

**Figure 2:**
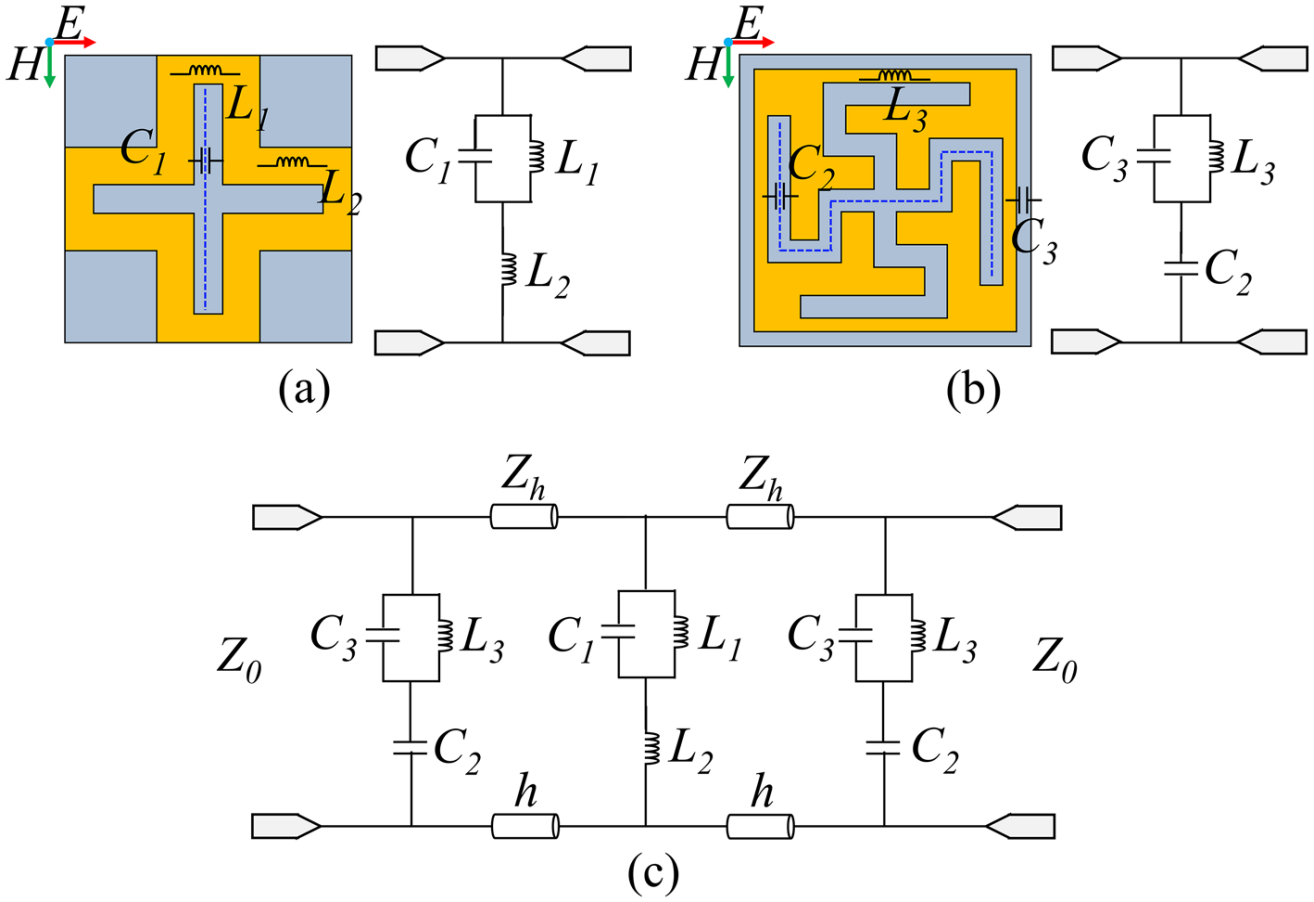
ECM under the normal incident waves, (**a**) ECM of middle layer, (**b**) ECM of top layer and bottom layer, (**c**) ECM of of the proposed FSS.

Here, *L*_*E*_ and *C*_*E*_ represent the equivalent inductance and capacitance of the components, respectively. Figure 2 illustrates the ECM of the proposed FSS structure established along the direction of the electric field. The ECM of the middle layer under normal incident waves is shown in Fig. 2a, consisting of a parallel LC circuit and a series inductance. The equivalent inductance of the grid structure is represented by *L*_1_ and *L*_2_, while the equivalent capacitance of the cross-shaped gap embedded in the grid structure is denoted as *C*_1_. Since the top and bottom layers of the FSS structure have the same unit structure, the circuit configuration of the top and bottom layers is identical, as depicted in Fig. 2b. This ECM comprises a parallel LC circuit and a series capacitance. The equivalent inductance of the capacitive patch edge is denoted as *L*_3_, *C*_2_ represents the equivalent capacitance of the curved cross gap, and *C*_3_ represents the equivalent capacitance between the two capacitive patches. The remaining components of the structure, such as the dielectric substrate and air layer, can be modeled as short transmission lines with a characteristic impedance of *Z*_0_ = 377 Ω and *Z*_*h *_= 377/$\sqrt {\varepsilon_{r} }$Ω, as shown in Fig. 2c.

**Figure 3:**
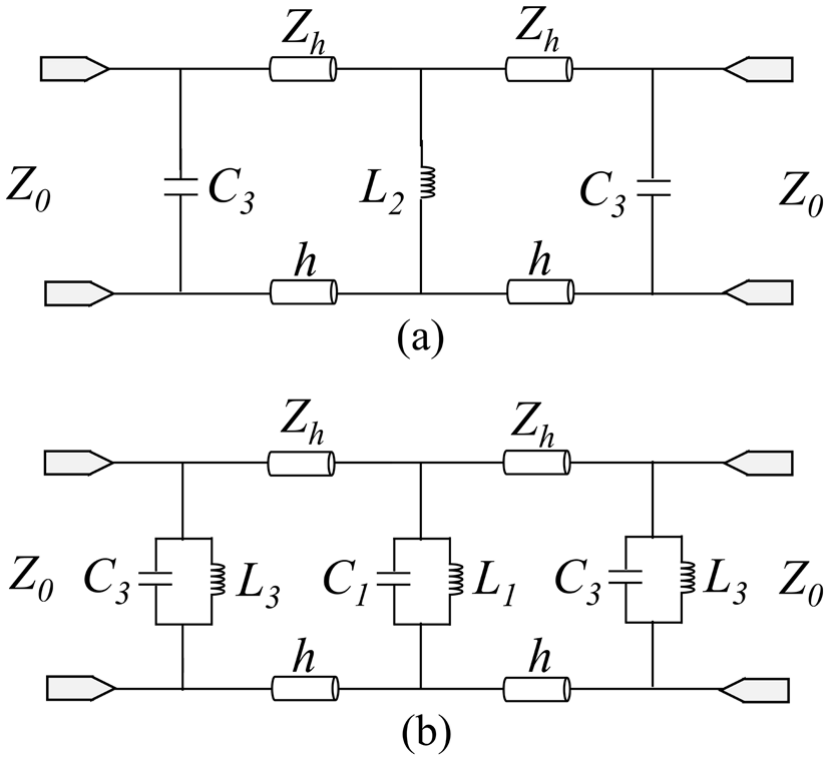
Simplified model of each passband, (**a**) First passband, (**b**) Second passband.

According to the equivalent circuit shown in Fig. 2c, the resonant frequencies of the parallel resonators *L*_1_*C*_1_ and *L*_3_*C*_3_ primarily determine the frequency of the second passband, while the frequency of the first passband is mainly determined by the inductance *L*_2_, capacitance *C*_3_, and the length *h* of the transmission line. When the frequency separation between the two passbands is sufficiently large, the influence of the parallel LC resonator can be neglected. Consequently, a simplified ECM for the first passband can be obtained, as depicted in Fig. 3a. This circuit is a second-order coupled-resonator bandpass filter (BPF) with inductive coupling. The center frequency of the passband is:

2 $$f_{1} = \frac{1}{{2\pi \sqrt {(C_{3} + \frac{{\varepsilon_{0} \varepsilon_{r} h}}{2})(L_{2} + \mu_{0} \mu_{r} h)} }}$$

The principal resonant mechanism for the second passband is the parallel resonator; therefore, inductance *L*_2_ and capacitance *C*_2_ have little effect. A simplified ECM for the second passband is shown in Fig. 3b, which consists of three parallel LC resonators divided by a brief transmission line of length *h*. As a result, the following formula can be used to calculate the second passband's center frequency:

3 $$f_{2} = \frac{1}{{2\pi \sqrt {\left[ {C_{1} C_{2} + \frac{{\varepsilon_{0} \varepsilon_{r} h}}{2}\left( {C_{1} + C_{2} } \right)} \right]\left( {\frac{{L_{1} + L_{3} + \mu_{0} \mu_{r} h}}{{C_{1} + C_{2} }}} \right)} }}$$

According to transmission line theory, the equivalent capacitance and equivalent inductance of the proposed FSS element can be estimated using the following formulas^41^ :

4 $$L_{1} \approx \mu_{0} \mu_{r} \frac{W}{2\pi }\log \left( {{1 \mathord{\left/ {\vphantom {1 {\sin \frac{{\pi \left( {P - l} \right)}}{2W}}}} \right. \kern-0pt} {\sin \frac{{\pi \left( {P - l} \right)}}{2W}}}} \right)$$

5 $$L_{2} \approx \mu_{0} \mu_{r} \frac{P}{2\pi }\log \left( {{1 \mathord{\left/ {\vphantom {1 {\sin \frac{{\pi \left( {W - W_{1} } \right)}}{4P}}}} \right. \kern-0pt} {\sin \frac{{\pi \left( {W - W_{1} } \right)}}{4P}}}} \right) - L_{1}$$

6 $$C_{1} \approx \varepsilon_{0} \varepsilon_{r} \frac{2l}{\pi }\log \left( {{1 \mathord{\left/ {\vphantom {1 {\sin \frac{{\pi W_{1} }}{2l}}}} \right. \kern-0pt} {\sin \frac{{\pi W_{1} }}{2l}}}} \right)$$

7 $$L_{3} \approx \mu_{0} \mu_{r} \frac{D}{2\pi }\log \left( {{1 \mathord{\left/ {\vphantom {1 {\sin \frac{\pi S}{{4D}}}}} \right. \kern-0pt} {\sin \frac{\pi S}{{4D}}}}} \right)$$

8 $$C_{2} \approx \varepsilon_{0} \varepsilon_{r} \frac{A}{\pi }\log \left( {{1 \mathord{\left/ {\vphantom {1 {\sin \frac{{\pi W_{2} }}{2A}}}} \right. \kern-0pt} {\sin \frac{{\pi W_{2} }}{2A}}}} \right)$$

9 $$C_{3} \approx \varepsilon_{0} \varepsilon_{r} \frac{D}{\pi }\log \left( {1/\sin \frac{{\pi \left( {P - D} \right)}}{2D}} \right)$$

The effective dielectric constant of the substrate is denoted as *ɛ*_*r*_, and the effective magnetic constant is denoted as *μ*_*r*_. The calculated values* A* and *S* are derived from the given dimensions using the following equations: *A* = 2(*l*_1_ + *l*_2_ + *l*_3_) + *l*_4_-6*W*_2_, and* S* = *D*-*l*_4_-2(*l*_2_-*W*_2_)*.* Table 2 provides the specific numerical values for the circuit components.

**Table 2:** Relevant electromagnetic parameters of ECM.

Parameter	Values	Parameter	Values
*L*_1_	0.067 nH	*L*_2_	0.049 nH
*L*_3_	0.171 nH	*L*_4_	0.171 nH
*C*_1_	0.165 pF	*C*_2_	0.108 pF
*C*_3_	0.077 pF	*C*_4_	0.108 pF
*C*_5_	0.077 pF	*Z*_*h*_	231.7 Ω

### Simulation results and discussion

**Figure 4:**
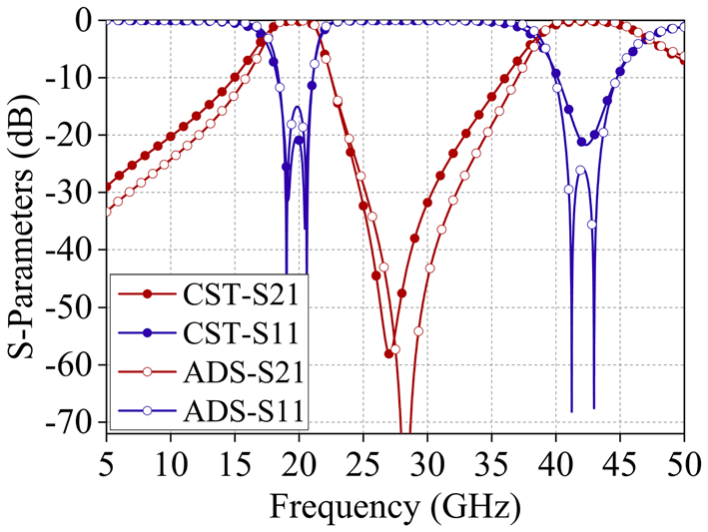
Comparison of the S-parameter curves obtained from ADS and CST simulations.

The performance of the designed dual-band FSS was simulated using electromagnetic simulation software. Additionally, the frequency response of the FSS's ECM was analyzed using ADS software. Figure 4 illustrates the simulated results of the FSS when subjected to the normal incidence of electromagnetic waves. The FSS design exhibits broadband transmission characteristics in the Ku and Ka frequency bands. At the edges of each passband, the electromagnetic response transitions rapidly from transmission to reflection. The results of the ADS circuit simulation and the CST full-wave simulation agree.

**Figure 5:**
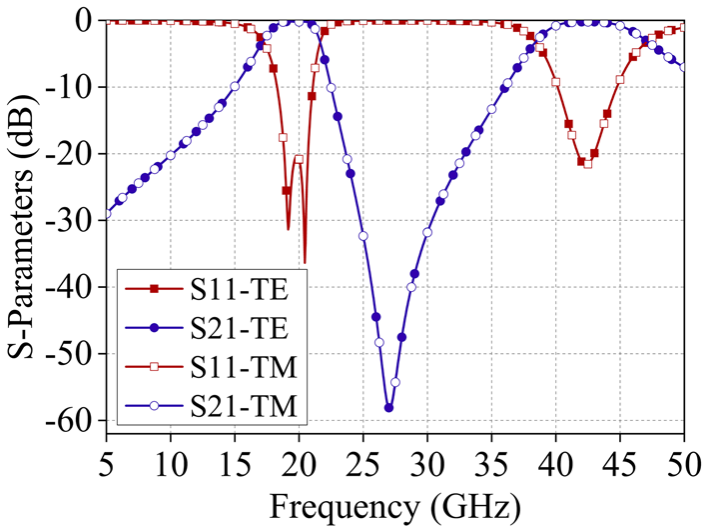
S-parameters obtained from CST full-wave simulation.

**Figure 6:**
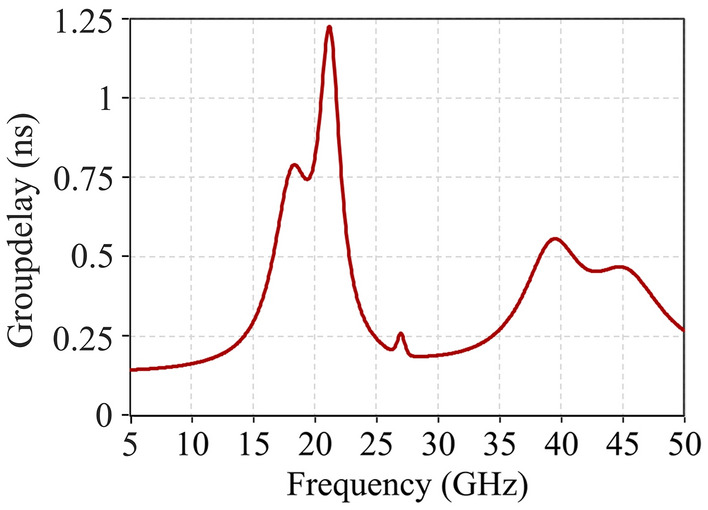
Group delay simulation result of the designed FSS.

Figure 5 displays the simulated transmission characteristics of the FSS under normal incidence of TE and TM polarized waves. The S-parameters for both polarizations demonstrate significant consistency, indicating that the dual-band FSS design is polarization-stable. The simulated − 3 dB transmission passbands span from 17.25 to 21.59 GHz and 38.51 to 47.05 GHz, with center frequencies of *f*_1_ = 19.42 GHz and *f*_2_ = 42.78 GHz, respectively. The group delay time of the proposed FSS for incident electromagnetic waves is given in Fig. 6.

**Figure 7:**
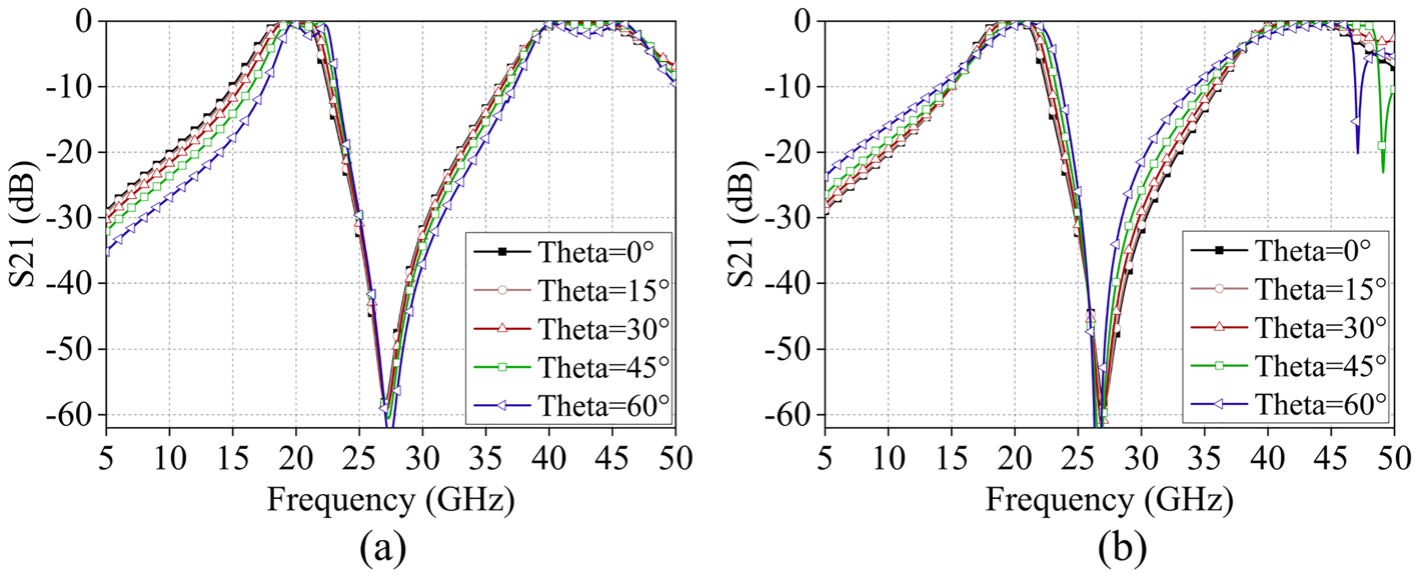
Simulated transmission coefficient of the proposed FSS at oblique incidences, (**a**) TE polarization, (**b**) TM polarization.

The simulated reflection and transmission coefficients of the designed FSS under the oblique incidence of TE and TM polarizations are depicted in Fig. 7. It is evident that the proposed FSS demonstrates a reliable transmission performance of up to 60° for both TE and TM polarizations. With the increase in incident angle, both passbands consistently maintain high transmittance. In TE polarization, there is a slight increase in insertion loss within both passbands, and the first passband exhibits a slight frequency shift towards higher frequencies. Meanwhile, for TM polarization, the transmission characteristics remain relatively stable.

**Figure 8:**
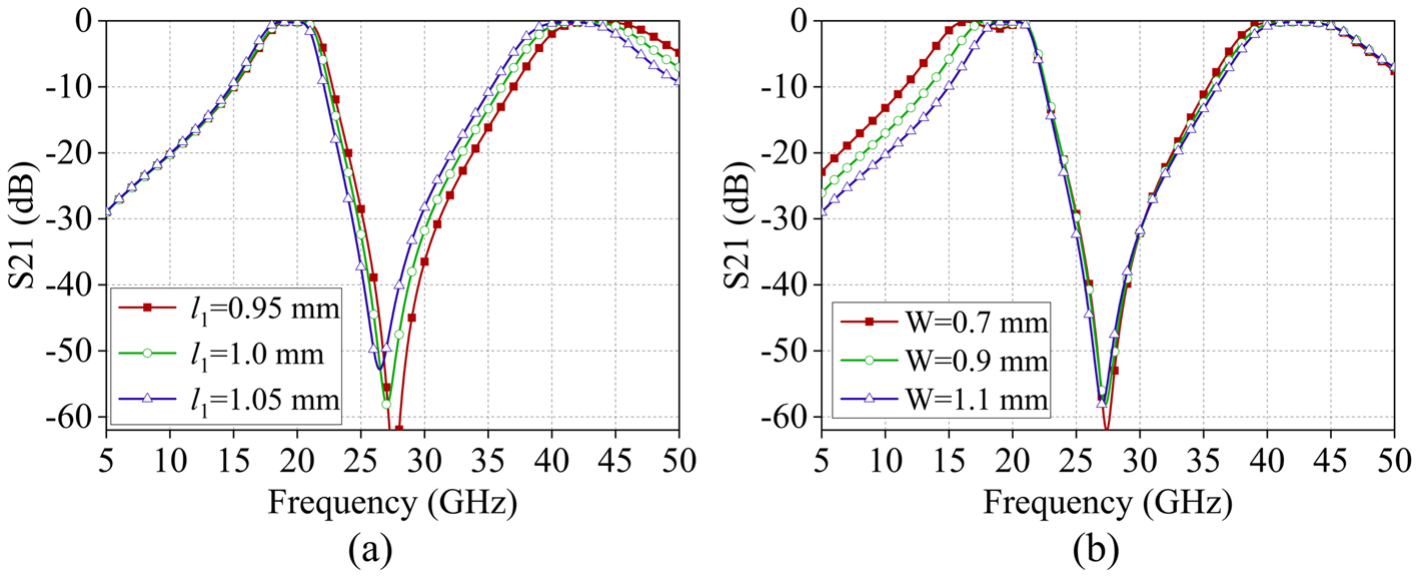
Effect of different geometric parameters on the transmission coefficient, (**a**) Side length *l*_1_, (**b**) Width *W*.

Furthermore, we examined the impact of key geometric parameters of the FSS structure on its transmission characteristics. Figure 8a illustrates that increasing the side length *l*_1_ of the upper and lower curved cross slots causes the center frequency *f*_2_ of the second passband to shift towards the lower frequency, while* f*_1_ remains relatively unchanged. This shift can be attributed to the increased *l*_1_, which leads to a rise in capacitances *C*_2_ and *C*_3_, according to the ECM. Consequently, based on the equation, the frequency shift for *f*_2_ will move to a lower frequency. Similarly, Fig. 8b demonstrates that increasing the width *W* of the intermediate layer grid results in a decrease in inductance *L*_3_, causing *f*_1_ to shift towards higher frequencies while *f*_2_ remains relatively unchanged. Based on the analysis mentioned above, it is clear that by adjusting the dimensions of the FSS structure, the position of either the second or first passband can be selectively tuned.

### Experimental validation

**Figure 9:**
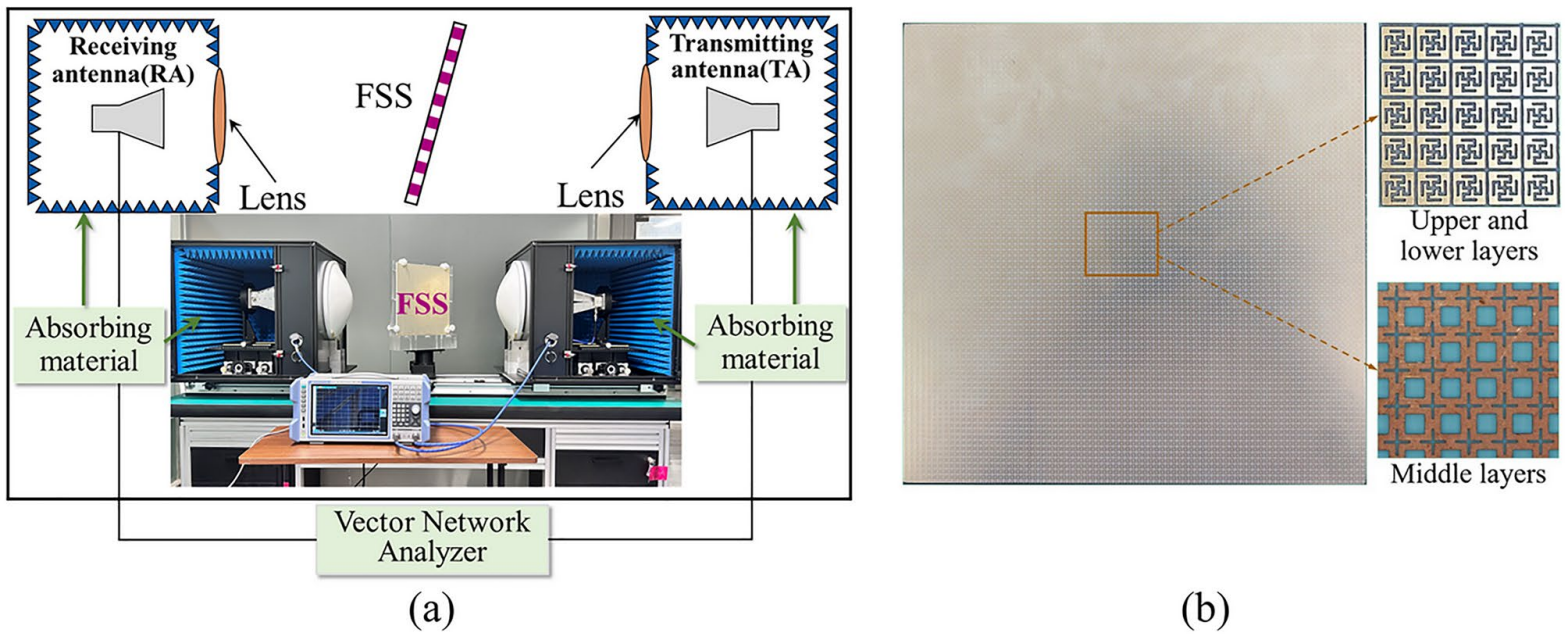
Photos of the measurement circumstance, (**a**) Photograph of the measurement setup, (**b**) Prototype of the manufactured FSS.

To validate the performance of the proposed dual-band FSS, a prototype sample was fabricated using printed circuit board (PCB) technology. Figure 9 showcases an image of the fabricated prototype sample of the dual-band FSS and the transparency testing system. The fabricated sample dimensions are 200.1 mm × 200.1 mm, consisting of 87 × 87 unit cells. The sample is constructed using a dielectric substrate of F4BM (*ε*_*r*_ = 2.65, *tanδ* = 0.0013). The complete testing system consists of a rotatable support frame, a vector network analyzer (VNA), two focusing lenses, and standard horn antennas, as depicted in Fig. 9a. The support frame is centrally located in the transmission and reflection paths of the two horn antennas, with electromagnetic lenses positioned between the support frame and the antennas. The two standard horn antennas are interconnected to the VNA via coaxial cables. To achieve accurate measurement outcomes, the testing is carried out in two phases: firstly, calibration is performed by assessing the transmission characteristics without the FSS sample, and secondly, the transmission characteristics are measured with the FSS sample inserted between the horn antennas. Subsequently, the obtained transmission coefficient is normalized to reduce the influence of the test setup and environmental factors. Additionally, considering the wide frequency range of the measurements, two sets of standard horn antennas are sequentially utilized, operating at 2–18 GHz and 18–50 GHz.

### Transmission performance

**Figure 10:**
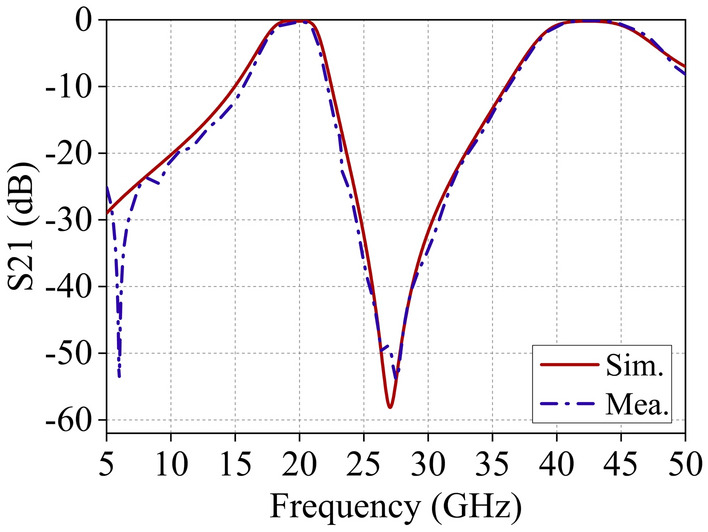
Comparison of FSS simulation and test results.

**Figure 11:**
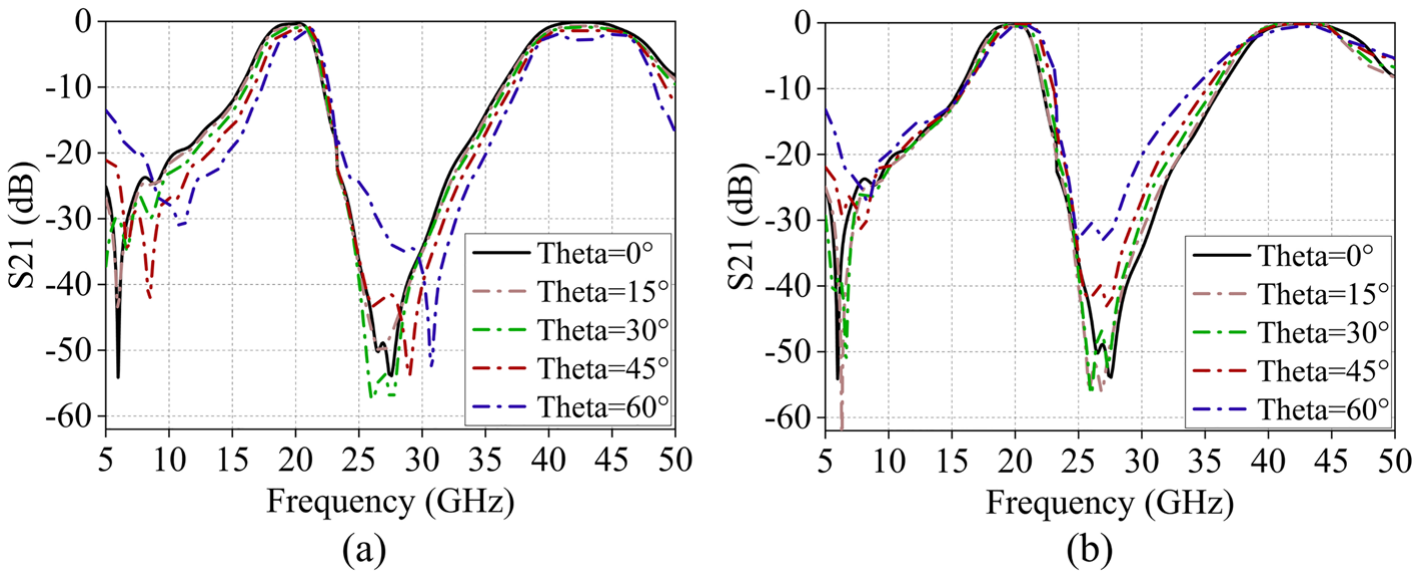
Test results of S21 curves at different incidence angles, (**a**) TE polarization, (**b**) TM polarization.

Figure 10 illustrates the transmission characteristics of the proposed FSS obtained from full-wave simulations and free-space testing. The simulated results closely match the test results, demonstrating dual-band broadband filtering properties centered at *f*_1_ = 19.42 GHz and *f*_2_ = 42.78 GHz. Both passbands exhibit second-order bandpass responses. Moreover, this structure demonstrates dual-band broadband transmission characteristics, with low insertion loss (0.34 dB and 0.24 dB) in-band and strong out-of-band suppression simultaneously. The roll-off rate ξ, defined as |(*α*_max_–α_min_)/(*f*_3dB_–*f*_10dB_)| (dB/GHz), quantifies frequency selectivity. A higher ξ indicates superior frequency selectivity^42,43^. Here, *α*_max_ and *α*_min_ denote the 10- and 3-dB attenuation points, while *f*_10dB_ and *f*_3dB_ represent their corresponding frequencies. The roll-off rate ξ at the lower and upper edges of the first passband is 3.4 and 8.6 dB/GHz, respectively, and at the lower and upper edges of the second passband is 3.0 and 2.4 dB/GHz. Furthermore, an analysis of the transmission characteristics of this structure at different incident angles is presented in Fig. 11. For both polarization modes, the insertion loss of the two passbands remains within − 3 dB as the incident angle varies from 0° to 60°. This indicates the excellent angle stability and polarization insensitivity of the proposed FSS structure.

A comparative analysis of operating frequency, bandwidth, insertion loss, and incident angle between the current study and existing designs is presented in Table 3. It is important to note that many existing FSS designs are limited to either microwave or millimeter-wave bands and lack the capability to operate effectively in both simultaneously. The proposed design demonstrates ample bandwidth, competitive insertion loss, and higher angular stability under oblique incidences of TE and TM modes with incident angles up to 60°. These attributes position the proposed FSS as a viable solution for dual-band radome applications in modern airborne detection radars operating across microwave and millimeter-wave frequencies. Additionally, it can be applied in satellite communication and filtering systems to address interference challenges between multiple modules and optimize radar system performance^8,9^.

**Table 3:** Comparison with recently published results.

References	Central frequency (GHz)	− 3 dB FBW	IL (0° )	Stability	Profile (*λ*_10_)	Periodicity (*λ*_10_)
^6^	27.9	27.2%	0.8 dB	45°	0.54	0.38
^16^	27.75	9.6%	1.6 dB	60°	0.103	0.25
^10^	28	6.72%	1.73 dB	50°	**—**	0.23
^7^	60	4.03%	2 dB	40°	0.06	0.49
^33^	8.5 /11.5	6.1%/4.2%	≤ 0.3 dB	60°	0.02	0.23
^34^	12.1 /15.05	5.8%/3.5%	0.9 dB/1.73 dB	40°	0.03	0.65
^24^	16.5/31	10%/7.14%	**—**	45°	0.1	0.26
^35^	10.6/24.1/42.5	46%/16.7%/21.4%	≤ 2 dB	< 30°	0.007	0.12
This work	19.42/42.78	22.4%/20%	0.34 dB/ 0.24 dB	60°	0.05	0.14

*λ_0_ is the free-space wavelength corresponding to the center frequency of the first passband.

The main contributions of this article can be summarized as follows:

1. The article introduces a novel technique for designing dual-band frequency-selective surfaces. This technique is founded on the specific topology of a second-order band-pass microwave filter and involves synthesizing its constituent elements using periodic structures with inductive, capacitive, or resonant-type surface impedances.
2. The design demonstrates adequate bandwidth, competitive loss, and increased angular stability under oblique incidences of TE and TM modes with incident angles up to 60° in the dual-band of microwave and millimeter wave frequencies.
3. A novel broadband second-order bandpass FSS based hybrid unit cell composed of resonant and nonresonant structures is proposed. The proposed FSS has second-order band-pass responses at each band of operation.

## Conclusion

This paper presents the design of a novel wideband dual-bandpass FSS for dual-band radar applications in the microwave and millimeter-wave bands. The proposed FSS offers significant advantages in terms of wide bandwidth, low insertion loss, excellent angular stability, and miniaturization capabilities. The center frequencies of the passbands are *f*_1_ = 19.42 GHz and* f*_2_ = 42.78 GHz, and the − 3 dB bandwidth is 4.34 GHz (17.25–21.59 GHz) and 8.54 GHz (38.51–47.05 GHz), respectively. Furthermore, to delve deeper into the operational principles of the FSS, an ECM of the overall structure was developed to analyze the impact of various parameters on the FSS’s performance. A prototype sample of the FSS is manufactured and subjected to testing. The experimental results are in good agreement with the simulation results, demonstrating excellent stability across different incident modes and angles.

## Data Availability

The raw/processed data are being used by ongoing research projects, thus these findings cannot be shared at this time. If necessary, please contact the corresponding author: meibi0323@163.com.
